# XGBoost and SHAP-Based Analysis of Risk Factors for Hypertension Classification in Korean Postmenopausal Women

**DOI:** 10.3390/bioengineering12060659

**Published:** 2025-06-16

**Authors:** Hojeong Kim, Mavlonbek Khomidov, Jong-Ha Lee

**Affiliations:** 1 Department of Biomedical Engineering, Keimyung University, Daegu 42601, Republic of Korea; hcathy@kmu.kr; 2 Department of Computer Engineering, Keimyung University, Daegu 42601, Republic of Korea; mavlonbek@stu.kmu.ac.kr

**Keywords:** hypertension, postmenopausal, eXplainable artificial intelligence, Shapley Additive exPlanations, machine learning

## Abstract

In postmenopausal women, the prevalence of hypertension increases sharply, emphasizing the importance of its prevention. This increased risk highlights the critical need for effective prevention strategies specifically designed for this population. To address this issue, the present study aimed to identify easily measurable risk factors that contribute to hypertension in postmenopausal women using explainable artificial intelligence (XAI) and machine learning (ML) techniques. This study conducted hypertension classification by analyzing health checkup data from 3289 postmenopausal Korean women aged 55–79 years, extracted from the 2022–2023 Korea National Health Insurance Service (KNHIS) database, using XGBoost, SVM and ANN. XGBoost was the most effective model (AUC: 92.12%, MCC: 0.71) in hypertension classification. Shapley Additive exPlanations-based feature importance identified age and waist circumference (WC) as the most important risk factors for hypertension. In this study, blood pressure increased with variations in WC, a modifiable risk factor. These findings suggest that WC should be managed more strictly to prevent hypertension in postmenopausal women.

## 1. Introduction

Hypertension can cause mortality and various complications such as cardiovascular disease (CVD), kidney disease, and metabolic syndrome [1]. According to a 2024 report by the Korean Society of Hypertension, 30% of Korean adults over the age of 20 had hypertension in 2022. In particular, the prevalence of hypertension is lower in women than in men, but it increases rapidly after menopause, becoming equal to or higher than that in men [2]. The risk of developing hypertension doubles after menopause, affecting approximately 75% of postmenopausal women in the United States [3]. Menopause is a natural and expected stage in a woman’s life. As the global population ages, approximately 25 million women enter menopause each year. By 2030, the number of women who are menopausal or postmenopausal is expected to reach 1.2 billion [4].

Estrogen deficiency in postmenopausal women leads to changes in the vascular system, making blood pressure regulation more difficult and increasing the risk of CVD [5]. However, prevention of hypertension is lacking, because it is hard to notice changes in abnormal blood pressure due to psychological distress. Additionally, in daily life, almost no one checks their blood pressure regularly. This is because traditional cuff-based monitors are not very convenient for everyday use. Since the incidence of hypertension in postmenopausal women is rapidly increasing worldwide, early prevention and effective blood pressure management are more critical than ever. We analyzed risk factors that can estimate hypertension in daily life more easily.

The objectives of this study are as follows:

- To analyze risk factors for hypertension in postmenopausal women using machine learning (ML) and explainable artificial intelligence (XAI) techniques.
- To identify factors that directly affect the development of hypertension by minimizing the confounding effects of other diseases in a group of postmenopausal women in whom all clinical parameters except blood pressure were within the normal range.
- To evaluate the effect of changes in waist circumference (WC), a modifiable risk factor, on blood pressure prediction using SHAP (Shapley Additive exPlanations), and to explore the potential for developing personalized health management strategies based on this analysis.

This study is the first to use ML and XAI algorithms to identify risk factors for hypertension in postmenopausal women and is expected to provide basic data for early control and reduction of the risk of hypertension in postmenopausal women.

## 2. Related Work

Numerous researchers have investigated various risk factors contributing to hypertension in postmenopausal women, aiming to identify the physiological, hormonal, and lifestyle-related changes that increase the risk of developing hypertension during this stage of life. In previous studies, researchers categorized menopausal status into several distinct stages. Their finding showed that women in the postmenopausal stage had significantly higher blood pressure levels compared to women in earlier stages [6,7]. Tao et al. [8] conducted a separate analysis of systolic and diastolic blood pressure among 346 postmenopausal women. Their results showed that the body mass index (BMI) was significantly associated with variations in systolic blood pressure (SBP), whereas diastolic blood pressure (DBP) changes were more closely related to alcohol consumption. Additionally, age, anti-hypertensive agents, hip circumference, and marital status were associated with both SBP and DBP.

The postmenopausal period is commonly linked to an increased risk of obesity; studies show that approximately 40% of women in this group are classified as overweight [9,10]. Cifkova et al. [11] found that BMI was the most influential predictor of blood pressure among the various factors analyzed in postmenopausal women. Khitan et al. [12] demonstrated a significant correlation between SBP, BMI, and WC in postmenopausal women. Zhang et al. [13] conducted a 20-year longitudinal study of 3436 normotensive Chinese adults and found that increases in WC were strongly linked to upward trends in blood pressure. Their results suggest that WC may be a more sensitive and predictive marker than BMI for early detection of hypertension risk. In a study involving 1422 pre- and postmenopausal Korean women, Park et al. [14] found a significant correlation between WC and SBP. This relationship remained robust even after adjusting for potential confounding factors such as age and BMI, suggesting that WC may serve as an independent predictor of elevated SBP in this population.

More recently, ML and XAI algorithms have been actively utilized for CVD research. In previous studies, Guarneros-Nolasco et al. [15] mainly identified major risk factors through ML in a public CVD dataset and emphasized the importance of algorithmic selection and performance evaluation across various classifiers. Shirley et al. [16] applied a SHAP-based XAI technique to develop a prediction model for CVD occurrence within the next 10 years. It demonstrated how SHAP values can enhance the interpretability of ML outputs and support clinical decision-making through transparent feature attribution.

While these studies primarily focused on evaluating model performance and improving explainability, this study extends the clinical utility of XAI not only for interpretability but also for refining preventive screening criteria in real-world practice, specifically targeting postmenopausal women. Unlike previous research that often analyzed data containing various complications, this study performed an analysis specialized for hypertension using data in which only blood pressure was an outlier and the remaining variables (BMI, WC, fasting glucose, total cholesterol, triglycerides, high-density lipoprotein cholesterol, low-density lipoprotein cholesterol, aspartate aminotransferase, alanine aminotransferase, gamma-glutamyl transpeptidase, creatinine) were within the normal range (Table 1). Through this, the influence of confounders was excluded, and only risk factors that increase hypertension were intensively analyzed. In particular, considering that WC in postmenopausal women has a great influence on the risk of hypertension, this study directly applied XAI to clinical prevention protocols and provided evidence supporting the need to revise the conventional WC cutoff of 85 cm to a lower threshold for hypertension risk management.

## 3. Materials and Methods

### 3.1. Dataset

#### 3.1.1. Study Population

**Figure 1:**
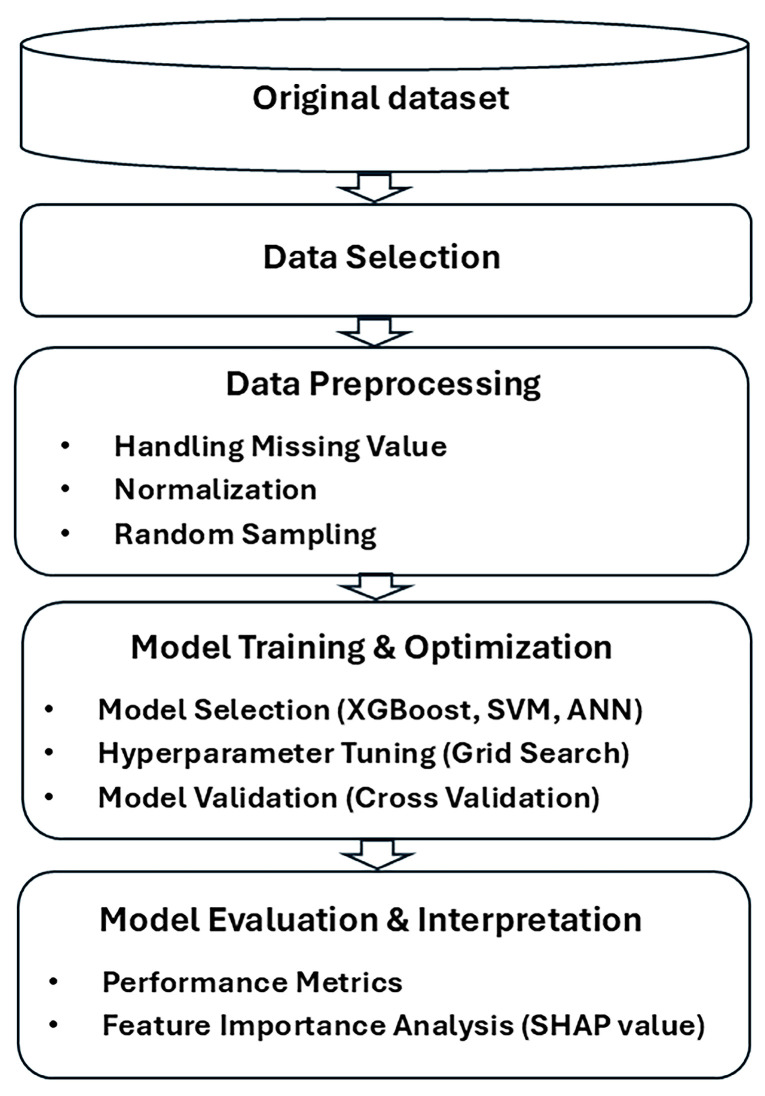
Methodology for the analysis of the hypertension classification using the 2022–2023 Korean National Health Insurance Service health examination dataset.

This study was a cross-sectional study conducted using data from the 2022–2023 Korea National Health Insurance Service (KNHIS), specifically the General Health Check-up and Transition Period Health Examination records. From the 2,000,000 participants extracted from the KNHIS 2022–2023 dataset, men (N = 1,030,336), participants younger than 55 or older than 79 (N = 400,840), and those with missing values (N = 389,548) were excluded. This study focused on postmenopausal women with normal blood test results, excluding participants (N = 176,037) whose blood test values exceeded the reference ranges, as specified in Table 1. As a result, the final dataset included 3289 women (23% hypertensives) aged 55 to under 80 years (Figure 1).

The analyzed variables included age, BMI, height, weight, WC, fasting glucose (BST), total cholesterol (TC), triglycerides (TG), high-density lipoprotein cholesterol (HDL-C), low-density lipoprotein cholesterol (LDL-C), hemoglobin (Hb), aspartate aminotransferase (AST), alanine aminotransferase (ALT), gamma-glutamyl transpeptidase (rGTP), and creatinine. The above variables are standard values of the regular KNHIS health checkup, and were used for this research purpose of identifying hypertension risk using available data. SBP and DBP, directly associated with the diagnosis of hypertension, were excluded from the ML analysis. Since SBP and DBP were used as criteria to define hypertension, they were excluded because they could cause data leakage when used as input variables in hypertension classification analysis, which would reflect the results as they are rather than predicting them, thus lowering the validity of the model. All measured values except SBP and DBP used the normal range values presented in the guideline provided by KNHIS (Table 1). This approach is designed to isolate the contribution of predictive factors for hypertension without interference from underlying comorbidities or extreme outliers. The statistical information for the variables used in this study is provided in Table 2.

#### 3.1.2. Definition of Variables

**Figure 2:**
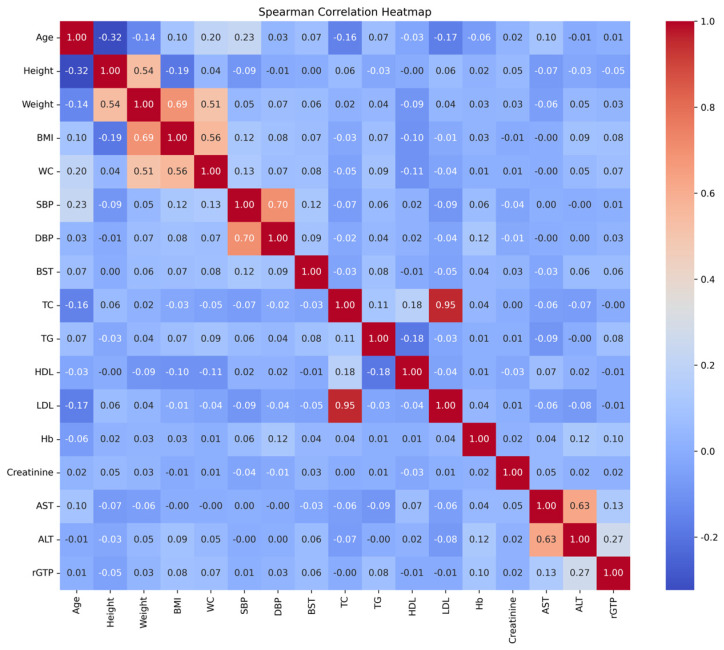
Heatmap of Pearson correlation coefficient for measured variables.

Participants were divided into normotensive and hypertensive groups according to their blood pressure levels. According to the 2022 Hypertension Treatment Guidelines for the Korean Society of Hypertension, hypertension is defined as SBP ≥ 140 mmHg or DBP ≥ 90 mmHg, with participants meeting this criterion classified as hypertensives. Participants with SBP < 90 mmHg and DBP < 60 mmHg, meeting the criteria for hypotension, were excluded from the study as they did not meet the definition of normal blood pressure. Normotensives were defined as all remaining participants who were neither hypertensive nor hypotensive. The target variable was the presence (hypertensives) or absence (normotensives) of hypertension. The independent variables comprised demographic factors such as age, anthropometric measurements including BMI, weight, height, and WC, and blood test results such as BST, TG, HDL-C, LDL-C, Hb, ALT, rGTP, and creatinine. Independent variables were blood tests and anthropometric tests performed in standard medical checkups, and all variables were included except those with a high correlation between variables. Comparisons between hypertensives and normotensives were performed using an independent two-sample *t*-test in SPSS 30.0 to evaluate the significance of the differences. The Pearson correlations between variables were visualized as a heatmap using JupyterLab version 4.0.11 with Python 3.12.4 (Figure 2).

### 3.2. Data Preprocessing

#### 3.2.1. Data Normalization

Normalization is essential for AI algorithms sensitive to data scales. In this study, normalization was performed using min–max scaling to adjust numeric features to a uniform range between 0 and 1 [17]. This method preserved the differences in value ranges while preventing information loss. Min–max scaling is mathematically expressed as follows:

$$X_{\mathrm{norm}}=\frac{X-X_{min}}{X_{max}-X_{min}}$$

where *X* is the original value, *Xmin* and *Xmax* are the minimum and maximum values of the feature, respectively, and *Xnorm* is the normalized value. This process ensured that no single feature disproportionately influenced the model due to its scale, enabling balanced and unbiased analyses.

The analyzed variables included age, BMI, height, weight, WC, BST, TC, TG, HDL-C, LDL-C, Hb, AST, ALT, rGTP, and creatinine. SBP and DBP, directly associated with the diagnosis of hypertension, were excluded from the ML analysis. The statistical information for the variables used in this study is provided in Table 3.

#### 3.2.2. Balancing Data

A combination of random oversampling and undersampling techniques was employed, resulting in a balanced 1:1 ratio between normotensives and hypertensives [18]. Random oversampling increases the number of samples in the minority class. Given that the generated data retains the same characteristics as the original data, it may lead to overfitting but reduces the risk of introducing noise. Undersampling is a technique utilized to selectively remove data from the majority class, which is a suitable method for continuous-variable data, making it an appropriate data balancing technique for this study. In addition, we used the Matthews Correlation Coefficient (MCC) to confirm the reliability and comprehensiveness of model performance evaluation.

### 3.3. Feature Importance Analysis

#### 3.3.1. Explainable Artificial Intelligence (XAI)

XAI emerged through the Defense Advanced Research Projects Agency’s Wireless Access in Infrastructure (WAI) program in 2016 to address interpretability challenges of black-box ML models [19]. XAI enhances the transparency and reliability of models by providing clear explanations of their decision-making processes. It identifies key variables used by complex AI models and clarifies their impact on decision-making, facilitating a deeper understanding of the processes and strengthening the credibility of research results. XAI techniques range from simple methods, such as feature importance analysis, to advanced approaches like Shapley Additive exPlanations (SHAP) values and Local Interpretable Model-agnostic Explanations (LIME). In this study, SHAP was employed to analyze feature importance in a hypertension prediction model.

#### 3.3.2. Shapley Additive exPlanations (SHAP)

The SHAP method, introduced by Lundberg and Lee, is based on the cooperative game theory and calculates Shapley values to quantify the individual contributions of each feature to the target, thereby determining feature importance [20]. This method addresses the black-box nature of ML models and explains how each feature influences the output, enhancing the interpretability of the model’s output. Various versions of SHAP have been developed to approximate SHAP values for different ML algorithms, including tree-based models and neural networks. In particular, SHAP values are well suited to analyzing their contribution to disease risk in clinical contexts, as they provide both global and local interpretability.

In this study, the TreeSHAP estimation methodology, specifically designed for tree-based approaches, was employed to determine the feature importance contributing to the hypertension classifier. TreeSHAP is not only more efficient but also provides the added advantage of evaluation interaction effects. Calculating feature importance using the SHAP methodology is more stable and consistent compared with methods like random forest. It enhances the interpretability of the hypertension classifier and provides deep insights into the dataset by clearly showing how specific features influence predictions. Using SHAP force plots, the local interpretation of a specific forecast is possible, for example, demonstrating how changes in the most important features, such as age and WC, contribute to hypertension. Thus, SHAP is a valuable, popular, and explainable AI method used across various fields, offering both local and global explanations by combining individual local explanations and comparing each data point’s prediction with the average prediction.

### 3.4. Performance Evaluations

**Figure 3:**
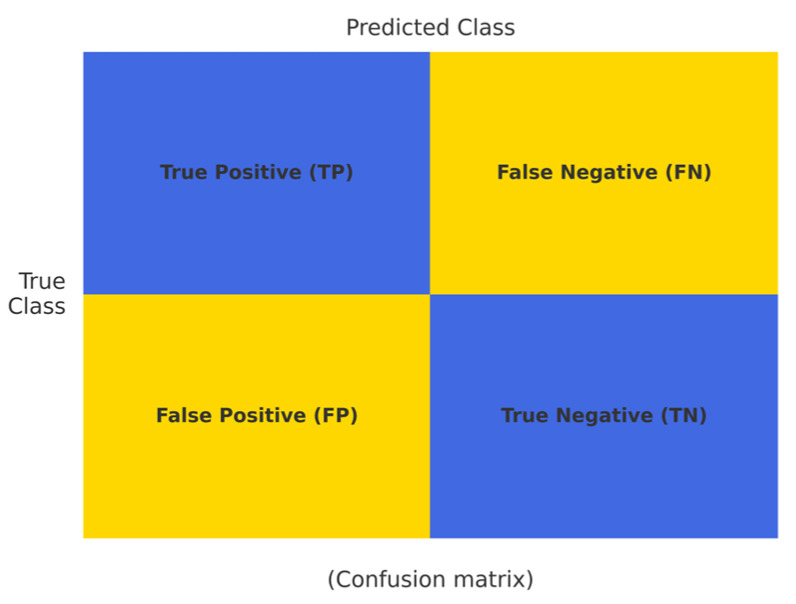
Confusion matrix for hypertension classification performance.

Hypertension classification performance was evaluated using accuracy, precision, sensitivity, specificity, F1-score, ROC-AUC and MCC. The confusion matrix (Figure 3) provides a detailed breakdown of true positive, true negative, false positive, and false negative rates, illustrating the classifier’s performance on the test dataset. The dataset was balanced between hypertensives and normotensive groups by combining oversampling and undersampling, but the MCC was additionally calculated to ensure the reliability of model performance. MCC quantifies the correlation between predicted and actual labels, and is an objective metric for evaluating the overall performance of each classification model. An MCC value of +1 indicates a perfect prediction, 0 indicates statistically insignificant prediction performance, and −1 indicates that the prediction results are completely opposite to the true class [21]. All analyses, including data preprocessing, model development, evaluation, and visualization, were conducted using Jupyter Lab version 4.0.11 with Python 3.12.4.

### 3.5. Algorithmic Enhancement

In this study, we employed 5-fold cross-validation [22] using Python’s sklearn.model_selection [23]. In this study, GridSearchCV provided by Python’s scikit-learn package was used for hyperparameter optimization. GridSearchCV systematically searches for specified hyperparameter combinations and repeatedly performs cross-validation for each combination to select the optimal combination. GridSearchCV, optimizing model parameters on 80% of the training data, while holding the remaining 20% for testing, to ensure model robustness. K-fold cross-validation is a crucial technique for utilizing all parts of the data effectively, to assess model generalization and prevent overfitting. GridSearch systematically explores and evaluates various hyperparameter combinations to optimize model performance (Table 3). This method is advantageous for ensuring the generalization performance of the model in that it can evaluate how consistently each parameter combination performs without overfitting.

## 4. Results

### 4.1. Baseline Characteristics and Correlation Analysis

The values for BMI, WC, BST, TC, TG, HDL-C, LDL-C, Hb, AST, ALT, rGTP, and creatinine were based on standardized health criteria (Table 1), provided by the National Health Insurance Service Database Open Data. Factors that could influence blood pressure, such as pre-existing conditions (e.g., metabolic disorders, kidney diseases, hyperlipidemia, and diabetes), were excluded. This study focused on identifying risk factors that directly affect blood pressure in women aged 55–79, while minimizing the influence of comorbidities.

Significant differences between normotensives and hypertensives were observed in the following factors: age (61.12 years vs. 63.58 years), BMI (21.72 kg/m^2^ vs. 22.10 kg/m^2^), height (154.03 cm vs. 152.86 cm), WC (74.11 cm vs. 75.52 cm), SBP (118.81 mmHg vs. 146.68 mmHg), DBP (72.03 mmHg vs. 86.20 mmHg), and BST (96.63 mg/dL vs. 99.37 mg/dL). The other factors were not statistically significant (Table 2).

To identify the correlation between variables, Pearson correlation coefficients were calculated. A correlation coefficient of 0.6 or higher was considered indicative of a strong correlation. Strong correlations were observed in the following variable pairs: TC-LDL (0.95), SBP-DBP (0.70), weight–BMI (0.69), and AST-ALT (0.63). Variables with strong correlations, including weight, AST and TC, were excluded from the ML analysis to avoid multicollinearity.

### 4.2. Hypertension Classification Performance

**Figure 4:**
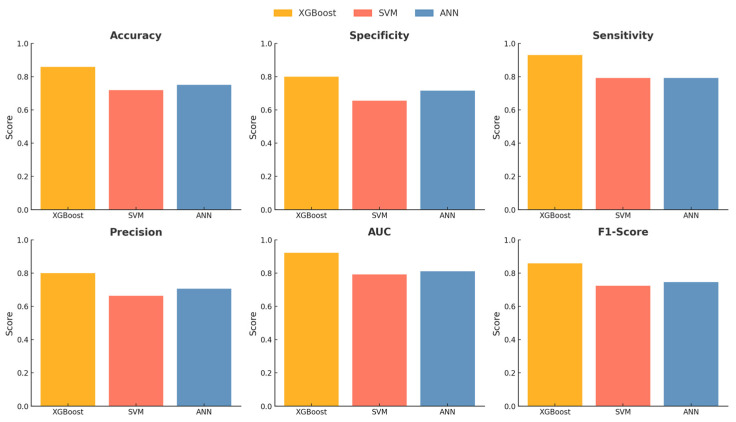
Comparison of hypertension prediction performance.

**Figure 5:**
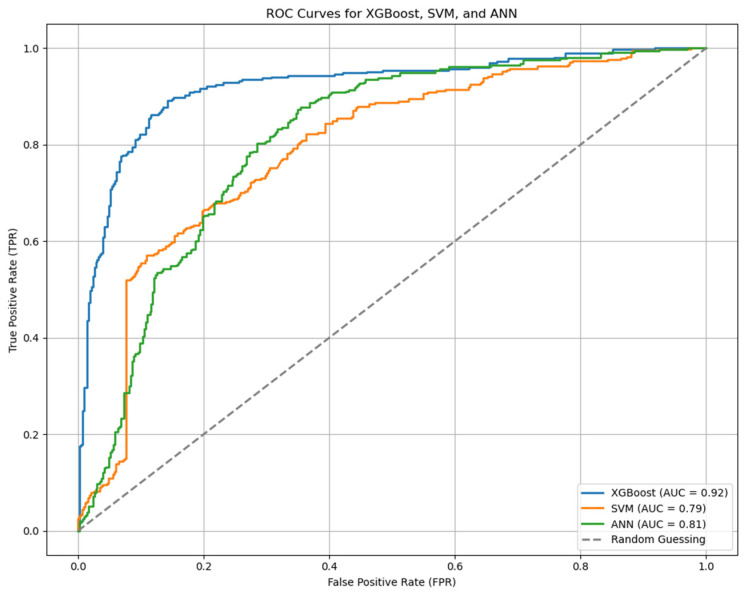
ROC curve for hypertension classification performance.

The ML analysis focused on hypertension classification performance, SHAP-based feature importance, and local insights for blood pressure prediction. For the hypertension classification performance analysis, SBP and DBP values, which could have the greatest impact, were excluded. The analysis was conducted using all 12 explanatory variables, based on importance ranking. Among the models, XGBoost demonstrated a relatively robust performance. XGBoost achieved the best performance with an accuracy of 84.73%, specificity of 78.09%, sensitivity of 92.43%, precision of 78.44%, AUC of 92.12%, F1-score of 84.86%, and MCC of 0.71 using all 12 explanatory variables (Table 4, Figure 4 and Figure 5).

**Figure 6:**
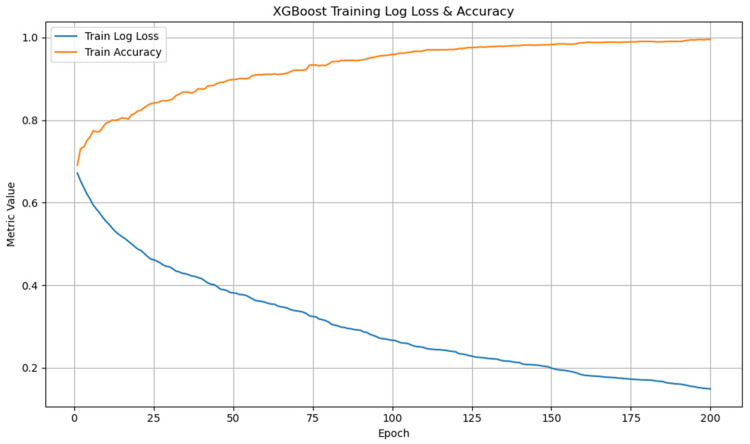
Log loss and accuracy changes during XGBoost training for hypertension classification.

In addition, to verify the stability and effectiveness of the training process, we tracked the changes in log loss and training accuracy during XGBoost training for over 200 epochs. As shown in Figure 6, the training log loss continuously decreased, while the training accuracy increased, eventually reaching a stable level of close to 99%. This shows that the model converged appropriately without overfitting or divergence.

### 4.3. SHAP-Based Feature Importance and Local Insights for Blood Pressure Prediction

**Figure 7:**
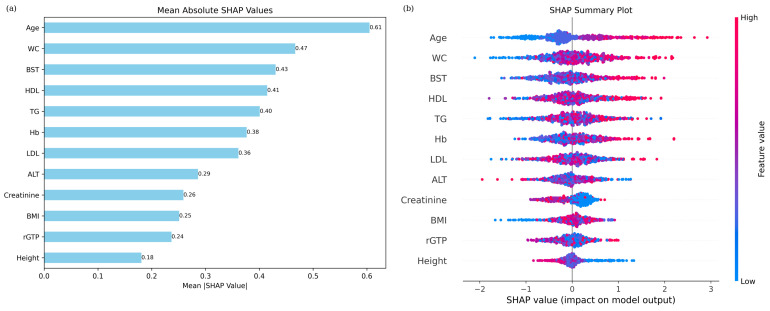
(**a**) SHAP feature importance based on mean absolute SHAP values: age and WC were the top-ranked features. (**b**) SHAP summary plot for hypertension classification based on XGBoost, showing the direction and magnitude of each feature’s impact. Red means higher and blue means lower impact on hypertension risk.

The SHAP-based feature importance analysis revealed the following ranking of variables: age (0.61), WC (0.47), BST (0.43), HDL-C (0.41), TG (0.40), Hb (0.38), LDL-C (0.36), ALT (0.29), creatinine (0.26), BMI (0.25), rGTP (0.24), and height (0.18) (Figure 7a).

The analysis revealed that age significantly contributes to hypertension, with its impact becoming more pronounced in older individuals. WC was identified as a critical factor influencing blood pressure, even though all measurements were below the threshold for abdominal obesity (85 cm). Large WC values were associated with a higher contribution to hypertension. Similarly, BST levels, even within the normal range, showed a greater contribution to hypertension as values increased, although diabetes status could not be determined due to the absence of medical history. HDL-C and LDL-C levels exhibited a tendency to increase hypertension risk when SHAP values were high; however, the trend was not distinctly clear. ALT did not show any clear trends in terms of their contributions to hypertension. Lower TG levels were associated with a reduced risk of hypertension, whereas higher levels were associated with an increased likelihood. Lower creatinine levels were associated with a high contribution to hypertension, and SHAP values for creatinine were densely distributed near zero. The overall effect of creatinine on the hypertension classification model seemed to be limited. BMI, even within the normal range (below 25), showed that lower values were linked to a reduced contribution to hypertension. Similarly, rGTP contributed to hypertension regardless of whether SHAP values were positive or negative, but no clear trend was observed. Height, on the other hand, revealed that shorter stature was associated with a higher contribution to hypertension, while taller height corresponded to a reduced contribution (Figure 7b).

**Figure 8:**
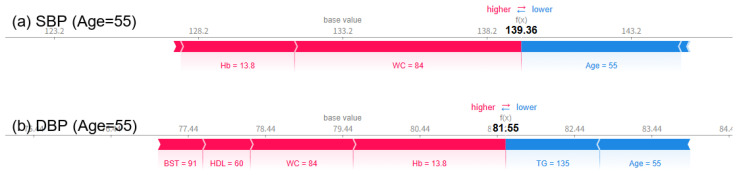
SHAP force plots for a 55-year-old woman showing a predicted SBP (**a**) of 139.36 mmHg and DBP (**b**) of 81.55 mmHg.

**Figure 9:**
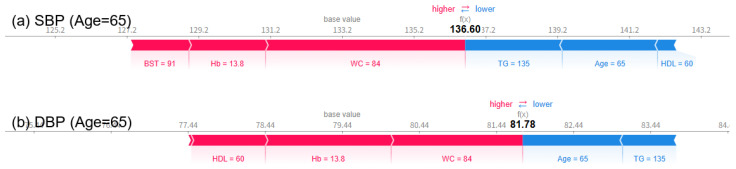
SHAP force plots for a 65-year-old woman showing a predicted SBP (**a**) of 136.60 mmHg and DBP (**b**) of 81.78 mmHg.

**Figure 10:**
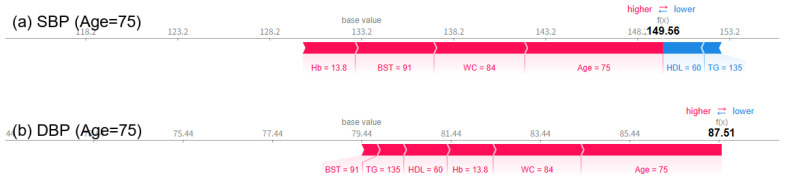
SHAP force plots for a 75-year-old woman showing a predicted SBP (**a**) of 149.56 mmHg and DBP (**b**) of 87.51 mmHg, exceeding hypertension thresholds.

Analysis using SHAP force plots visually shows how blood pressure predictions change with age, and clearly reveals the influence of key explanatory variables on the predictions. At age 55, SBP and DBP were predicted to be 139.36 mmHg and 81.55 mmHg (Figure 8), respectively, and at age 65, they were slightly lowered to 136.60 mmHg and 81.78 mmHg (Figure 9), respectively. However, at age 75, SBP rose significantly to 149.56 mmHg and DBP to 87.51 mmHg (Figure 10), respectively, exceeding the criteria for hypertension diagnosis. Along with this, pulse pressure (PP) also increased significantly from 57.81 mmHg at age 55 to 54.82 mmHg at age 65, to 62.05 mmHg at age 75. There was minimal change in blood pressure and PP in patients in their 50s and 60s, but both indicators showed a significant increase as they entered their 70s. The SHAP force plot visually decomposes and shows the contribution of each variable that constitutes the predicted value, with the red bars indicating factors that increase the predicted value (in the direction of hypertension), and the blue bars indicating factors that decrease the predicted value (in the direction of normal blood pressure). In particular, in the force plot of a 75-year-old (Figure 10), variables such as age, WC, BST, Hb, and HDL-C are all strongly represented in red, confirming that they cumulatively contributed to the increase in SBP and DBP. On the other hand, relatively low HDL-C levels and high TG levels did not contribute to lowering the predicted value, and rather, most variables worked in the direction of hypertension, resulting in a predicted value for hypertension that exceeded the diagnostic criteria. This is a representative example showing that major variables can interact and accumulate nonlinearly to have a strong effect on blood pressure prediction.

**Figure 11:**
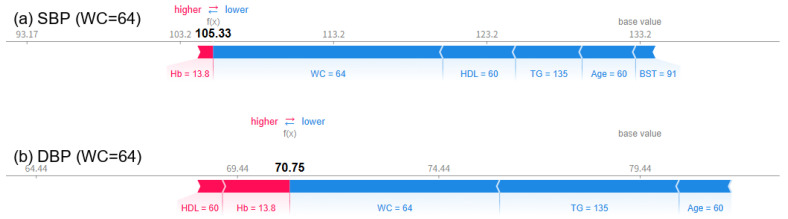
SHAP force plots for a case with WC = 64 cm, showing a predicted SBP (**a**) of 105.33 mmHg and DBP (**b**) of 70.75 mmHg.

**Figure 12:**
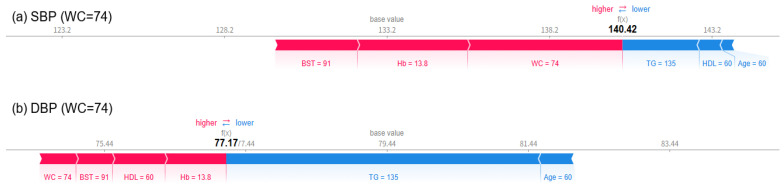
SHAP force plots for a case with WC = 74 cm, showing a predicted SBP (**a**) of 140.42 mmHg and DBP (**b**) of 77.17 mmHg. At this threshold, SBP exceeds the hypertension diagnostic criteria. This scenario suggests that WC near 74 cm marks a critical inflection point for hypertension risk.

**Figure 13:**
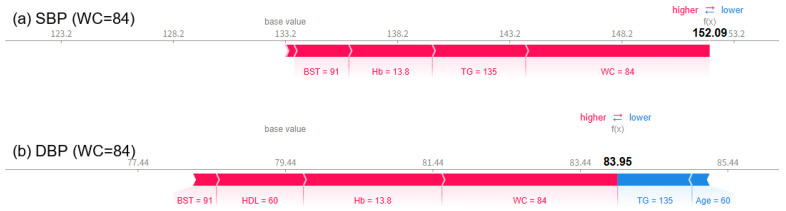
SHAP force plots for a case with WC = 84 cm, showing a predicted SBP (**a**) of 152.09 mmHg and DBP (**b**) of 83.95 mmHg. This reinforces the potential need to revise the conventional WC cutoff downward from 85 cm for early hypertension risk detection.

As a result of visually analyzing the effect of changes in waist circumference (WC) on blood pressure prediction using the SHAP force plot, it was confirmed that both SBP and DBP significantly increased as WC increased. When WC was 64 cm, the predicted SBP was 105.33 mmHg and DBP was 70.75 mmHg (Figure 11), which were within the normal range. At this time, variables such as TG, HDL-C, age, WC, and BST all appeared in blue and acted in the direction of lowering blood pressure. When WC increased to 74 cm, SBP increased to 140.42 mmHg and DBP increased to 77.17 mmHg (Figure 12), and in particular, it was confirmed that SBP exceeded the hypertension diagnostic criteria. From this point on, the red contributions of WC, Hb, BST, etc., increased noticeably, working towards increasing the predicted value. Finally, when WC reached 84 cm, the predicted SBP was 152.09 mmHg, DBP was 83.95 mmHg (Figure 13), with both indices clearly exceeding the hypertension range, and it was visually confirmed that variables such as BST, WC, Hb, and TG strongly contributed to the prediction of hypertension, appearing as thick and long red bars. PP also increased sharply from 34.58 mmHg when WC was 64 cm to 63.25 mmHg when WC was 74 cm and to 68.14 mmHg when WC was 84 cm. These results suggest that WC not only has an independent effect on blood pressure but also interacts nonlinearly with metabolic indicators such as BST, Hb, and TG to cause blood pressure elevation. In particular, since the risk of hypertension increases rapidly when WC exceeds 74 cm, it suggests the need to set a warning standard at a lower level than the existing 85 cm standard.

## 5. Discussion

This study used an XGBoost-based machine learning model to conveniently predict hypertension in postmenopausal women in daily life and to identify contributing factors. For accurate analysis, the analysis was conducted on subjects from the average menopausal age in their 50s [24] to those in their 70s, to which consistent hypertension criteria can be applied [25]. The hypertension classification model showed high predictive performance, and SHAP-based analysis confirmed that age, WC, BST, HDL-C, TG, Hb, LDL-C, ALT, creatinine, BMI, rGTP, and height contributed to hypertension in that order.

Age is the most important variable contributing to hypertension, but it is an unmodifiable factor [26]. In particular, in women, the prevalence of hypertension increases rapidly to a level similar to that of men (approximately 49%) in the group over 60 years of age, after the average menopause age [2]. In addition, as life expectancy increases, a healthy postmenopausal life has become increasingly important, emphasizing the need for early intervention and personalized prediction strategies to reduce the risk of hypertension, which causes various complications. For this reason, this study aimed to examine in depth the effect of the second most important variable, modifiable WC, on blood pressure.

Waist circumference is a representative indicator of abdominal obesity and is widely known as a risk factor for cardiovascular disease [27]. In Korean women, previous studies have shown that the risk of metabolic disease increases when the WC is over 85 cm, which has been adopted in current guidelines as a risk criterion for cardiovascular diseases [28]. The study that has been cited as evidence for the current cutoff criterion requires reevaluation from two perspectives.

First, there are limitations in using outdated databases and conventional statistical methods. Although previous studies have reported that lifestyle has a significant impact on blood pressure [1], current guidelines are still based on a database from around 2007. Hypertension prevalence continues to rise [2] despite advances in medical technology and medical facilities. This raises questions about whether current guidelines are still suitable. Conventional statistical approaches tend to suggest static thresholds, but this study introduces a dynamic cutoff approach by integrating ML and XAI technology that can directly show blood pressure changes according to WC changes through figures using the latest data, thereby confirming that SBP exceeded the hypertension diagnostic standard of 140 mmHg from the point where WC reached 74 cm. This was also found in prior research [29], supporting the validity of our analytic approach, but the model also provides clinically meaningful, individualized early intervention through the use of advanced modeling techniques.

Second, the previous study did not account for physiological differences associated with age. Similarly, many previous studies on risk factors for cardiovascular disease also presented universal standards that did not distinguish between age, sex, and race [30]. However, aging leads to a decline in physical functions, and hormonal effects differ between sexes, indicating that preventive guidelines should be tailored to specific population groups rather than applying universal standards. This principle also applies to racial differences. For example, the World Health Organization (WHO) recommends race-specific WC thresholds, setting the cutoff at 80 cm for Asian women [31]. Based on a meta-analysis of 18 studies conducted across 10 countries involving 155,122 individuals, Huxley et al. also found that the existing 85 cm cutoff value did not adequately reflect the risk of diabetes in Asian women [29]. These findings suggest that a lower threshold, particularly 80 cm, may be more appropriate for Asian women. In Japan, the national standard of 90 cm has been questioned by women, and it has been argued that flexible criteria should be set to take into consideration age and body type [32]. A study on the prediction of hypertension in black populations in West African and Caribbean island countries reported that WC thresholds differed across countries. In particular, the optimal WC criterion for predicting hypertension was 71.5 cm for Nigerian women [33], suggesting that the criteria for abdominal obesity differ significantly across populations.

This highlights the need to consider temporal, racial, gender, and age differences when establishing diagnostic criteria for hypertension risk associated with central obesity. Therefore, this study analyzed factors contributing to hypertension using the latest data on Korean postmenopausal women, and reflects the core principles of P4 medicine—‘Preventive’, ‘Personalized’, ‘Participatory’, and ‘Predictive’—which are future-oriented medical paradigms proposed by Leroy Hood [34].

Other variables include BST, which is closely related to metabolic diseases and insulin resistance through fasting blood sugar levels, and in this study, it showed a significant contribution to raising blood pressure along with WC. TG showed a positive association with hypertension. The trends of TG and BST were consistent with the cumulative effects of metabolic factors suggested in previous studies. HDL-C has also been suggested to be related to hypertension at both low and high levels [35], but in this study, the SHAP value of HDL-C had a relatively small contribution; thus, it was not a strong predictor. In the case of LDL-C, many previous studies have reported that LDL-C contributes more to vascular diseases such as atherosclerosis [36]. In contrast, WC has been reported to contribute more directly to the risk of hypertension [37]. Height is a relatively less-noticed variable, but it has been shown that shorter height tends to have a somewhat higher contribution to blood pressure. In this way, this study has addressed the limitations of existing statistical techniques that were expressed only in numbers by providing practical insights into clinical applicability and preventive approaches through interpretable AI-based analysis that quantitatively and visually confirms the individual contributions of various metabolic indicators to blood pressure.

In addition, this study offers insights from two key perspectives by using normal reference values for all biomarkers except blood pressure. First, since hypertension is closely related to various complications such as metabolic syndrome, kidney disease, and hyperlipidemia, this study minimized confounding effects by controlling for those conditions. This allowed for a more focused exploration of direct risk factors for hypertension. Second, whereas traditional threshold-based interpretations tend to assume that values within the normal range indicate no risk, this study quantitatively visualized the contribution of such biomarkers to hypertension risk using SHAP values. This highlights the concept of a ‘silent risk’ [38], where even clinically normal biomarker levels can contribute to disease progression without overt symptoms. These findings offer meaningful evidence for early-stage preventive interventions well before hypertension is clinically diagnosed.

These findings highlight the importance of early lifestyle interventions based on identified risk factors. A recent systematic review found that combined exercise training significantly reduced blood pressure in postmenopausal women, whereas aerobic exercise alone had limited effects [39]. Furthermore, long-term lifestyle interventions focusing on diet and physical activity have been shown to reduce abdominal fat accumulation during the menopausal transition [40]. These studies highlight the practical value of early, personalized risk factor identification, such as WC and metabolic markers, to design effective prevention strategies.

This study did not reflect lifestyle factors such as medication use, sodium intake, physical activity, and stress index due to the limitations of using open data. In addition, as the study focused exclusively on women from a single country, the generalizability of the findings to other ethnic and regional groups is limited. However, previous studies conducted in various countries have suggested WC thresholds below 85 cm [29,32,33]. Although the exact standard may vary depending on population characteristics, the methodology employed in this study holds strong potential for extrapolation, as it is based on analyzing contributions to the universally relevant physiological indicator of blood pressure.

## 6. Conclusions

This study analyzed the risk factors for hypertension in 3289 postmenopausal women aged 55–79 years, using health checkup data from 2022 to 2023. XGBoost achieved the best performance (AUC: 92.1%; MCC: 0.71) for hypertension classification. In this study, we highlight the important role of age and WC in predicting hypertension risk by integrating XAI and ML. While these results are consistent with previous studies, this study provides new insights by quantitatively visualizing the dynamic impact of each variable on blood pressure using SHAP values and identifying “silent risk” even within the normal biomarker range.

These results support that more refined blood pressure thresholds may be warranted for postmenopausal women. They also underscore the importance of adopting healthy dietary habits and maintaining consistent physical activity as essential strategies for hypertension prevention. Moreover, the proposed model demonstrates potential for personalized risk profiling, indicating that XAI approaches could serve as valuable tools in clinical decision-making and early intervention planning. To enhance the robustness and practical relevance of this model, it is necessary to validate these findings in more diverse populations and incorporate lifestyle-related and longitudinal data in future studies.

## Figures and Tables

**Table 1:** Reference ranges based on standardized health criteria provided by the Korean National Health Insurance Service (NHIS).

Measured Value	Reference Range
Body Mass Index (BMI)	<25 kg/m^2^
Waist Circumference (WC)	>85 cm
Fasting Glucose (BST)	80–130 mg/dL
Total Cholesterol (TC)	150–250 mg/dL
Triglyceride (TG)	30–135 mg/dL
High-Density Lipoprotein Cholesterol (HDL-C)	30–65 mg/dL
Low-Density Lipoprotein Cholesterol (LDL-C)	70–169 mg/dL
Hemoglobin (Hb)	12.5–15.5 g/dL
Aspartate Aminotransferase (AST)	0–40 IU/L
Alanine Aminotransferase (ALT)	0–40 IU/L
Gamma-Glutamyl Transpeptidase (rGTP)	8–35 IU/L
Creatinine	0.8–1.7 mg/dL

**Table 2:** Comparison of demographic information between normotensive and hypertensive patients.

	Normotensive		Hypertensive		*p* Value
	(N = 2487)		(N = 752)		
Age (years)	61.12	±5.334	63.58	±6.152	<0.001
BMI ^1^ (kg/m^2^)	21.72	±1.865	22.10	±1.708	<0.001
Height (cm)	154.03	±5.530	152.86	±5.700	<0.001
Weight (kg)	51.59	±5.328	51.68	±4.956	0.660
WC ^2^ (cm)	74.11	±5.512	75.52	±5.104	<0.001
Systolic BP (mmHg)	118.81	±11.492	146.68	±10.577	<0.001
Diastolic BP (mmHg)	72.03	±8.229	86.20	±8.851	<0.001
Fasting glucose (mg/dL)	97.63	±10.019	99.37	±10.709	<0.001
TC ^3^ (mg/dL)	195.55	±25.646	193.67	±25.922	0.080
TG ^4^ (mg/dL)	88.13	±25.687	90.78	±24.377	0.012
HDL-C ^5^ (mg/dL)	55.15	±6.980	55.46	±6.992	0.276
LDL-C ^6^ (mg/dL)	122.59	±24.882	119.79	±25.382	0.007
Hemoglobin (g/dL)	13.51	±0.680	13.56	±0.699	0.053
AST ^7^ (mg/dL)	25.05	±5.375	24.93	±5.496	0.618
ALT ^8^ (U/L)	19.05	±6.188	18.86	±6.301	0.469
rGTP ^9^ (U/L)	17.74	±5.690	17.87	±5.851	0.580
Creatinine (mg/dL)	0.86	±0.084	0.86	±0.094	0.473

^1^ BMI: body mass index; ^2^ WC: waist circumference; ^3^ TC: total cholesterol; ^4^ TG: triglycerides; ^5^ HDL-C: high-density lipoprotein cholesterol; ^6^ LDL-C: low-density lipoprotein cholesterol; ^7^ AST: aspartate aminotransferase; ^8^ ALT: alanine aminotransferase; ^9^ rGTP: gamma-glutamyl transpeptidase (*p*-values refer to the comparison between normotensive and hypertensive patients).

**Table 3:** Parameter configuration of ML models for hypertension prediction.

Algorithm	Parameters Settings
XGBoost ^1^	max_depth = 6, n_estimators = 200, learning_rate = 0.1
SVM ^2^	c = 10, kernel = rbf, gamma = scale
ANN ^3^	activation = relu, solver = adam, hidden_layer_sizes = (50, 50)

^1^ XGBoost: eXtreme Gradient Boosting; ^2^ SVM: support vector machine; ^3^ ANN: artificial neural network.

**Table 4:** Performance of hypertension classification (all values in %, except MCC).

Classifier	Accuracy	Specificity	Sensitivity	Precision	AUC	F1-Score	MCC ^1^
XGBoost	84.73	78.09	92.43	78.44	92.12	84.86	0.71
SVM	65.71	60.84	71.35	61.11	72.47	65.84	0.32
ANN	75.09	71.56	79.19	70.60	81.03	74.65	0.51

^1^ MCC: Matthews Correlation Coefficient, which ranges from −1 to 1.

## Data Availability

https://www.data.go.kr/data/15007122/fileData.do accessed on 30 August 2024.

## Abbreviations

The following abbreviations are used in this manuscript:

ALT	Alanine aminotransferase
ANN	Artificial neural network
AST	Aspartate aminotransferase
BMI	Body mass index
BST	Fasting glucose
CVD	Cardiovascular disease
DBP	Diastolic blood pressure
Hb	Hemoglobin
HDL-C	High-density lipoprotein cholesterol
LDL-C	Low-density lipoprotein cholesterol
ML	Machine learning
PP	Pulse pressure
rGTP	Gamma-glutamyl transpeptidase
SBP	Systolic blood pressure
SHAP	Shapley Additive exPlanations
SVM	Support vector machine
TC	Total cholesterol
TG	Triglyceride
WC	Waist circumference
XAI	Explainable artificial intelligence
XGBoost	eXtreme Gradient Boosting
